# How Did Zika Virus Emerge in the Pacific Islands and Latin America?

**DOI:** 10.1128/mBio.01239-16

**Published:** 2016-10-11

**Authors:** John H.-O. Pettersson, Vegard Eldholm, Stephen J. Seligman, Åke Lundkvist, Andrew K. Falconar, Michael W. Gaunt, Didier Musso, Antoine Nougairède, Remi Charrel, Ernest A. Gould, Xavier de Lamballerie

**Affiliations:** a Department of Infectious Disease Epidemiology and Modelling/Molecular Biology, Domain for Infection Control and Environmental Health, Norwegian Institute of Public Health, Oslo, Norway; b Department of Medical Biochemistry and Microbiology (IMBIM), Zoonosis Science Center, Uppsala University, Uppsala, Sweden; c St. Giles Laboratory of Human Genetics of Infectious Diseases, The Rockefeller University, New York, New York, USA; d Department of Microbiology and Immunology, New York Medical College, Valhalla, New York, USA; e Departmento de Medicina, Universidad del Norte, Barranquilla, Colombia; f London School of Hygiene and Tropical Medicine, London, United Kingdom; g Pôle de Recherche et de Veille sur les Maladies Infectieuses Émergentes, Institut Louis Malardé, Tahiti, French Polynesia; h UMR Emergence des Pathologies Virales (EPV: Aix-Marseille Université-IRD 190-INSERM 1207-EHESP), Marseille, France; i Institut Hospitalo-Universitaire Méditerranée Infection, APHM Public Hospitals of Marseille, Marseille, France

## Abstract

The unexpected emergence of Zika virus (ZIKV) in the Pacific Islands and Latin America and its association with congenital Zika virus syndrome (CZVS) (which includes microcephaly) and Guillain-Barré syndrome (GBS) have stimulated wide-ranging research. High densities of susceptible *Aedes* spp., immunologically naive human populations, global population growth with increased urbanization, and escalation of global transportation of humans and commercial goods carrying vectors and ZIKV undoubtedly enhanced the emergence of ZIKV. However, flavivirus mutations accumulate with time, increasing the likelihood that genetic viral differences are determinants of change in viral phenotype. Based on comparative ZIKV complete genome phylogenetic analyses and temporal estimates, we identify amino acid substitutions that may be associated with increased viral epidemicity, CZVS, and GBS. Reverse genetics, vector competence, and seroepidemiological studies will test our hypothesis that these amino acid substitutions are determinants of epidemic and neurotropic ZIKV emergence.

## OPINION/HYPOTHESIS

Zika virus (ZIKV) was first described in the African forests, where it circulates between nonhuman primates and sylvatic mosquitoes (1). More than 60 years after its discovery, fewer than 20 human infections had been reported. The first ZIKV epidemic occurred in Yap, Federated States of Micronesia, Pacific, in 2007 (2). Based on a serological survey, 73% of the inhabitants were infected. ZIKV then disappeared epidemiologically until a large outbreak occurred in French Polynesia (FP) in 2013 to 2014. The outbreak was the first in which congenital Zika virus syndrome (CZVS), Guillain-Barré syndrome (GBS), and non-vector-borne transmission (materno-fetal, sexual, and posttransfusion) occurred (1, 3). Retrospectively, cases of microcephaly were reported in the offspring of 1% of women calculated to have been infected in their first trimester of pregnancy during the FP outbreak (4). Subsequently, ZIKV continued to spread in the Pacific region (1) and emerged in the Americas in 2015. The emergence was associated with a dramatic increase in microcephaly (5–7), a manifestation of the congenital Zika virus syndrome (CZVS), leading WHO to declare a global health emergency. Concomitantly, the number of infected visitors returning from the Pacific and Latin America to their homelands in North America, Europe, Asia, and Australasia was increasing, thus extending the risk for ZIKV in areas in which the *Aedes* ZIKV-competent mosquito is present.

Two ZIKV lineages have been described: African and Asian. Strains that emerged in the Pacific Islands and Latin America belong to the Asian lineage. In late 2015, a ZIKV outbreak due to an Asian lineage strain was also associated with cases of microcephaly. It occurred on Cape Verde off the coast of Africa (1). These data suggest that the severe neurological complication of ZIKV infections (GBS and CZVS) are associated with the strains that emerged in French Polynesia and subsequently spread to the Pacific Islands and Latin America and back to Africa at least to a coastal island.

How did ZIKV emerge from its sylvatic forest existence to cause major epidemics throughout the Pacific and the Americas? Similar to dengue virus (DENV), ZIKV had the potential to adapt from a sylvatic cycle involving sylvatic mosquitoes and nonhuman primates to an urban cycle involving urban/suburban mosquitoes and humans (1).

One possibility is that the emergence of ZIKV is simply a consequence of the increasing global human population, increasing population of competent mosquito vectors, increasing urbanization, and increasing global transportation of commercial goods. Lack of ZIKV-specific population immunity was almost certainly a contributing factor to the emergence of ZIKV. Additionally, the possibility of immune enhancement arising from cross-reactions with related viruses such as dengue virus cannot be ignored (8). However, we share the view of Musso and Gubler that genetic changes are the most likely explanation for the dramatic emergence and neuroinvasiveness of ZIKV (1). This concept is supported by observations that genetic changes associated with vector specificity and epidemic potential in chikungunya virus (9) and increased virogenesis in West Nile virus (10) have been reported. Accordingly, to study the possible effect of nonsynonymous mutations in the open reading frame and changes in the 5′ and 3′ untranslated regions (UTRs), we investigated the evolution of ZIKV through analysis of an extensive range of complete ZIKV genomic sequences. We identify amino acid changes that arose progressively throughout the viral genome as the virus emerged out of Africa and gradually dispersed across Asia, the Pacific, and Latin America and the more restricted changes that occurred in the untranslated regions. Importantly, we highlight and discuss specific amino acid substitutions that are directly associated with the appearance of the altered epidemiological and neurotropic characteristics of epidemic Pacific/Latin American ZIKV.

### Methods.

To explore the temporal evolution of ZIKV, representative and robust complete ZIKV sequences available as of 29 May 2016 were retrieved from GenBank (http://www.ncbi.nlm.nih.gov/genbank). At the time, only one isolate was available from French Polynesia (FP). To study the possibility that some FP isolates had the mutation M/T2634V first documented in the Latin American strains, an additional 13 isolates from various times and places in the FP outbreak were sequenced in house. The in-house next-generation sequencing was performed with the Ion PGM sequencer (Thermo, Fisher Scientific), and analyses were conducted with the CLC Genomics Workbench software. RNA was extracted from serum or cell supernatant medium using the EZ1 Mini virus 2.0 kit and the EZ1 advanced XL machine (both from Qiagen). Complete virus genomes were amplified in 3 or 12 fragments using specific sets of primers with the Superscript III one-step reverse transcription-PCR (RT-PCR) Platinum TaqHifi kit (Thermo, Fisher Scientific).

Two alignments were constructed using Mafft v.7.266, keeping the reading frame consistent with amino acid positions: one alignment (see Data Set S1 in the supplemental material) contained only Asian, Pacific, and Latin American ZIKV (*n* = 67 sequences), and the other alignment (see Data Set S2 in the supplemental material) also included African ZIKV (*n* = 84 sequences). In addition, separate alignments were constructed for the 5′- and 3′-UTR regions (see Data Sets S3 and S4 in the supplemental material) for those isolates for which the UTRs were available. Evolutionary rates were estimated using BEAST 1.8.3. After model testing, all analyses were run with the SRD06 codon-partitioned model using the general time reversible (GTR) nucleotide substitution model with gamma distribution, a strict molecular clock with a continuous-time Markov chain (CTMC) prior, and a Bayesian skyline coalescent tree prior with a piecewise-constant demographic model. To ensure mixing of individual chains and sufficient effective sample size (>100), each data set was run three times for 100 million generations, sampling every 10,000 generations. After discarding 10% burn-in for each run, consensus files for each data set were produced using LogCombiner and TreeAnnotator (BEAST package). Consensus trees were then viewed and annotated in FigTree 1.3.1 (http://tree.bio.ed.ac.uk/software/figtree/). All computations were performed at the CIPRES web portal (11). The numbers of unique stepwise amino acid changes in the different clades were checked visually and compared in the combined African and non-African alignments using AliView v1.18 (12).

### Results.

**FIG 1:**
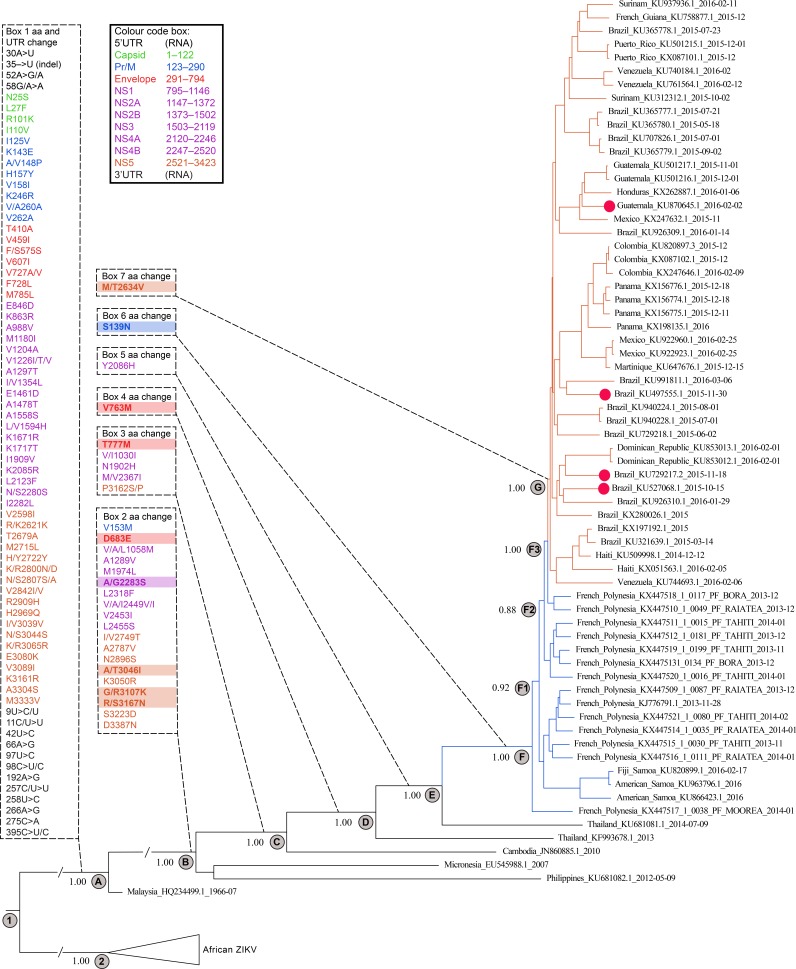
ZIKV phylogeny of African-Asian/Pacific and Latin American virus isolates, including mapping of amino acid substitutions. Dashed boxes show positions of amino acid substitutions relative to the open reading frame of ZIKV NC_012532.1, and dashed lines indicate the branch positions in the tree at which amino acid substitutions occurred. Amino acid substitutions of specific interest are highlighted in the boxes. The 5′- and 3′-UTR positions refer to the number of nucleotide positions before the start codon and after the stop codon, respectively. For clarity, the tree displays all Pacific virus branches in blue and all Latin American virus branches in red. Red circles highlight virus sequences that were retrospectively associated with CZVS. Posterior probability support values were identical except for node F1, which were 0.92 and 0.98, respectively, for the analyses that included or excluded the African lineages (Fig. 1, nodes 1 to 2 and A to G). Accession numbers for African genomes used in one of the analyses but not shown in the figure are as follows: HQ234498.1, DQ859059.1, KU720415.1, LC002520.1, NC_012532.1, AY632535.2, KU963573.1, KF268949.1, KF268948.1, KF268950.1, KU963574.1, HQ234501.1, KU955595.1, KU955592.1, KU955591.1, KF383116.1, and KX198134.1.

Two dated phylogenetic trees were constructed, and they produced identical topologies for the named key nodes. The first tree (Fig. 1) displays the African viruses in a collapsed format and also includes Asian, Pacific, and Latin American ZIKV isolates (Fig. 1). For the second tree (not shown), the African viruses were excluded. The boxes in Fig. 1 connected by dashed lines to the tree identify all amino acid substitutions that occurred beyond each branch (identified as nodes A to G). The 9 highlighted amino acids identified in boxes 2, 3, 4, 6, and 7 identify substitutions that will be discussed in the context of epidemic emergence of ZIKV and GBS/microcephaly. The conclusions drawn from the tree suggest that the African and Asian strains diverged from their ancestor around 1834 (95% highest posterior density interval [HPD], November 1814 to August 1852 [formatted throughout by year-month as “1814-11 to 1852-08”]) (node 1). The most recent common ancestor of the sylvatic African ZIKV emerged around 1889 (95% HPD, 1879-03 to 1898-01) (node 2). Furthermore, our analyses and supporting data extend the accumulating evidence (13, 14) that the Asian ZIKV emerged around 1946 (95% HPD, 1942-04 to 1953-10) based on the analysis, including African and Asian viruses, or around 1966 (95% HPD, 1964-02 to 1966-07), based on analysis including only Asian viruses. Importantly, none of the identified substitutions (Fig. 1, box 1) was accompanied by a change in the sylvatic tendency of the Asian strain isolated in Malaysia.

The first recorded dispersion of ZIKV out of Africa and Asia was to Yap Island (node B). This resulted in an explosive outbreak of ZIKV infection involving 73% of the immunologically naive population. However, as far as is known, this strain did not disperse further. Six years later, another epidemic Asian strain was identified in FP, documenting for the first time an association with microcephaly and GBS (3, 4, 15, 16). Based on the available phylogenetic data, it was considered possible that an FP strain was the source of the Latin American outbreaks of ZIKV disease (17). These authors identified 5 amino acid substitutions in the ZIKV prM protein that led them to predict major structural change in this protein. Nevertheless, the tree (Fig. 1) shows that 4 of these substitutions (at amino acid positions 143, 148, 157, and 158 of the prM protein) occurred in the Malaysian strain of ZIKV, which, in common with African ZIKV, caused only sporadic infections. The fifth of the prM substitutions, 153, did not occur until after node A, at which point all isolates, with the exception of the Philippine strain, were associated with epidemic spread.

Node B also identifies the point beyond which the ZIKV E protein receptor VGD changed to VGE via the amino acid substitution D683E. In general, the viral receptor plays an essential role in epidemic spread. This concept is supported by studies with a genetically engineered dengue 2 virus (DENV2) receptor loop (VEPG), which show a direct correlation between the receptor sequence and the ability of DENV-2 both to infect mammalian cells and to be transmitted by *Aedes aegypti* (18). Two additional and potentially important E protein amino acid substitutions, T777M (TM2; node C) and V763M (TM1; node D), were identified. These amino acids associate with the transmembrane (TM) region of infected cells and may play an active role in maintaining stability of the pH-induced trimeric E protein and also in mediating membrane fusion (19).

However, the Philippine and Micronesian isolates retained the African TM1 and TM2 amino acids (V763 and T777). The Cambodian isolate (node C) retained the African V763 TM1 amino acid but acquired the Asian 777M TM2 amino acid. The Thailand virus isolate identified by node D retained the Asian 777M and acquired the Asian amino acid substitution 763M and the second Thailand isolate identified by node E, retained the Asian 777M and 763M but in addition acquired Y2086H (Fig. 1). Thus, the Malaysian, Micronesian, Philippine, Cambodian, and Thai viruses may represent the final progressive evolutionary stages that endow ZIKV with epidemicity before the critical substitution that appears to coincide with emergence of neurotropic ZIKV in FP.

The first appearance of a French Polynesian virus in the tree is identified by node F. Node F1 identifies a branch with 3 Samoan viruses and 6 FP viruses, and node F2 identifies another branch with 5 FP viruses. Node F3 distinguishes a branch with 2 more FP viruses, which are the most closely related FP viruses to the Latin American viruses, identified by node G. Thus, nodes F, F1, F2, and F3 define 14 FP and 3 Samoan viruses that evolved in FP/Samoa over a period of less than 3 years. Note that nodes F1 to F3 are distinguished only by synonymous and sequence-specific nonsynonymous mutations.

Node F identifies the evolutionary point of emergence of epidemic neurotropic ZIKV, and this coincides precisely with the appearance of the amino acid substitution S139N in both the French Polynesian and Samoan isolates (Fig. 1, nodes F and F1). Recognition of the presence of ZIKV in human semen also coincides with the appearance of the S139N substitution. However, this may reflect more intensive study of ZIKV after its discovery in FP (20).

Amino acid 139 is in the ZIKV M protein, a transmembrane (TM) protein. While the function of this amino acid has not been defined for ZIKV, in DENV2 the equivalent amino acid is involved in the acid pH-mediated viral surface maturation process (21). Thus, all ZIKV isolates from FP onwards possess a VGE receptor sequence via the substitution D683E, the E protein TM1/TM2 substitutions (V763M/T777M), and the closely associated M protein TM modification (S139N).

Node G defines the point at which the substitution M/T2634V arises. Thus, the tree shows clearly that GBS and microcephaly, which were first reported in FP, arose following the S139N substitution in the ZIKV M protein. None of the FP isolates had the M/T2634V substitution found in the Latin American isolates. Although it will require further verification, the substitution most likely arose after ZIKV escaped the Pacific. Whether or not the M/T2634V substitution contributed to the recognized increased incidence of microcephaly in Latin America compared with the Pacific is a matter for conjecture until further relevant virus isolates become available or experimental evidence is obtained.

Did the five unique amino acid substitutions defined above—i.e., D683E (box 2), T777M (box 3), V763M (box 4), S139N (box 6), and M/T2634V (box 7)—have an impact on mosquito vector competence of epidemic ZIKV and increased ZIKV neurotropicity? These questions will be investigated using reverse genetics methods combined with vector competence and seroepidemiological studies.

The acquisition of four additional amino acid substitutions, including one in the NS4B protein (A/G2283S) and three in the NS5 protein (A/T3046I, G/R3107K, and R/S3167N), distinguishes African/Malaysian from other non-African ZIKVs (Fig. 1, box 2). These may have perturbed the intracellular interferon type I pathway (22). Additionally, as components of the viral replicative complex, these unique substitutions may contribute to enhanced viral replication (21, 23).

Comparative analysis of the 5′ and 3′ termini that form secondary structures in the untranslated regions (UTRs) of the ZIKV genome revealed differences between African and all other ZIKV isolates studied (Fig. 1, box 1; see Data Sets S3 and S4; see also Fig. S1 and S2). However, since all of these changes preceded the dispersion of ZIKV to both Micronesia and separately the Pacific region further east, they are unlikely to have been primary determinants of ZIKV epidemicity, although they may make a supplementary contribution. Another factor, not revealed by current analyses, is the influence of defective interfering virus particles. In DENV, such particles have been associated with increased epidemic potential (24).

Estimated mean evolutionary rates (MERs) and times to most recent common ancestor (tMRCA) based on strict molecular clock estimates, using trees that include or exclude African viruses, are also presented (Table 1; see Data Sets S1 and S2 in the supplemental material). Estimated MERs for the tree that included African viruses (6.30 × 10^−4^; 95% HPD, 5.5 × 10^−4^ to 7.1 × 10^−4^) were lower than those for the tree with African viruses excluded (1.20 × 10^−3^; 95% HPD, 1.1 × 10^−3^ to 1.3 × 10^−3^). Divergence of African and Asian ZIKV from a most recent common ancestor occurred around 1834 (95% HPD, 1814-11 to 1852-08). Subsequently there was a significant increase in the mean evolutionary rate of the Pacific/Latin American variants of ZIKV, an observation consistent with marked increase in virus transmission and turnover accompanying the massive island and urban outbreaks. tMRCA estimates indicate that the introduction of ZIKV into the Americas (Fig. 1, node G) occurred in November 2013 (95% HPD, 2013-10 to 2014-02). Although the timing is not significantly different from December 2013 (95% HPD, 2013-08 to 2014-04) of a previous estimate (13), the window of introduction is narrowed.

**TABLE 1:** Estimated times of origin and evolutionary rates of Zika viruses^a^

Node	Only Asian genomes^b^		African and Asian genomes^c^	
	tMRCA (yr-mo)	95% HPD (yr-mo)	tMRCA (yr-mo)	95% HPD (yr-mo)
1, African ZIKV/Asian ZIKV			1834-08	1814-11–1852-08
2, African ZIKV			1889-02	1879-03–1898-01
A, Malaysia 1966/other ZIKV	1966-01	1964-02–1966-07	1948-03	1942-04–1953-10
B, Philippines-Micronesia/other ZIKV	2001-09	2000-08–2002-09	1994-05	1991-08–1996-11
C, Cambodia/other ZIKV	2004-11	2003-10–2005-10	1999-02	1997-12–2001-04
D, Thailand/other ZIKV	2008-01	2007-02–2008-11	2003-10	2001-11–2005-06
E, Thailand/other ZIKV	2010-04	2009-07–2011-02	2007-03	2005-10–2008-08
F, PI/PI-American ZIKV	2013-02	2012-10–2013-05	2011-12	2011-03–2012-07
F1, PI/PI-American ZIKV	2013-05	2013-02–2013-07	2012-04	2011-08–2012-10
F2, PI/PI-American ZIKV	2013-06	2013-04–2013-11	2012-07	2012-01–2012-12
F3, PI/American ZIKV	2013-10	2013-08–2013-11	2012-10	2012-03–2013-02
G, American ZIKV	2013-11	2013-10–2014-02	2012-11	2012-05–2013-04

a tMRCA, time to most recent common ancestor; MER, mean evolutionary rate; HPD, highest posterior density interval; PI, Pacific Islands.

b MER, 1.20 × 10^−3^; 95% HPD, 1.09 × 10^−3^ to 1.31 × 10^−3^.

c MER, 6.30 × 10^−4^; 95% HPD, 5.48 × 10^−4^ to 7.10 × 10^−4^.

Our phylogenetic and comparative amino acid analyses revealed four unique amino acid substitutions: one (D683E [box 2]) was located in the viral receptor site, two (T777M [box 3] and V763M [box 4]) were located in the viral envelope protein, and the final but possibly the most critical one (S139N [box 6]) was located in the M protein. These amino acids clearly play important roles in virus attachment entry, release, or maturation of the virus during the infectious process. Thus, three unique amino acid substitutions directly associated with virus infection and transmission efficiency occurred just prior to the emergence and widespread dispersal of the highly epidemic and neurotropic ZIKV. The appearance of the membrane protein substitution S139N coincided precisely with the appearance of GBS and CZVS in FP. While this does not prove cause and effect, it appears to be more than a coincidence. Another unique substitution, in the NS5 protein of ZIKV (M/T2634V [box 7]) was identified in all Latin American viruses and may also impact neurotropicity since the relative rate of CZVS increased significantly in Brazil compared with FP.

Other substitutions in the prM and E glycoproteins could either be compensatory or could alter virus characteristics, such as hydrophilicity and charge. As reported for DENV mutants, the ZIKV mutants show efficiency for producing neurovirulence (25) and adsorption-mediated endocytosis (AME). (AME depends on electrostatic/hydrophobic interactions between the virion and cell surface [26].)

The emergence of ZIKV and its widespread dispersion throughout the tropics and subtropics have undoubtedly been influenced by increasing human mobility, urbanization, and failure to reduce the spread and expanding population density of ZIKV *Aedes* species vectors. The recent discovery that ZIKV is significantly more thermostable than DENV may also contribute to its success as an epidemic virus through its ability to reproduce to higher levels of viremia and to resist inactivation under unfavorable environmental conditions (27).

The use of reverse genetics, vector competence studies, and seroprevalence analyses to gain an understanding of the epidemic potential of ZIKV will enable the validity of our observations and hypotheses to be tested both *in vitro* and *in vivo.*

### Accession number(s).

The sequences of the additional French Polynesian isolates have been submitted to GenBank under accession no. KX447509 to KX447521.

## SUPPLEMENTAL MATERIAL

Data Set S1  Nucleotide alignment in fasta format of coding regions of Asian, Pacific, and Latin American ZIKV isolates. Download Data Set S1, TXT file, 0.6 MB

Data Set S2  Nucleotide alignment in fasta format of all Zika isolates. Download Data Set S2, TXT file, 0.8 MB

Data Set S3  Nucleotide alignment in fasta format of 5′ UTR of Zika isolates. Download Data Set S3, TXT file, 0.1 MB

Data Set S4  Nucleotide alignment in fasta format of 3′ UTR of Zika isolates. Download Data Set S4, TXT file, 0.1 MB

Figure S1  Alignment of 5′ UTRs of Zika virus isolates in PDF format. Download Figure S1, PDF file, 0.4 MB

Figure S2  Alignment of 3′ UTRs of Zika virus isolates in PDF format. Download Figure S2, PDF file, 1.5 MB
