# Erratum for Pettersson et al., “How Did Zika Virus Emerge in the Pacific Islands and Latin America?”

**DOI:** 10.1128/mBio.00386-18

**Published:** 2018-03-20

**Authors:** John H.-O. Pettersson, Vegard Eldholm, Stephen J. Seligman, Åke Lundkvist, Andrew K. Falconar, Michael W. Gaunt, Didier Musso, Antoine Nougairède, Remi Charrel, Ernest A. Gould, Xavier de Lamballerie

**Affiliations:** a Department of Infectious Disease Epidemiology and Modelling/Molecular Biology, Domain for Infection Control and Environmental Health, Norwegian Institute of Public Health, Oslo, Norway; b Department of Medical Biochemistry and Microbiology (IMBIM), Zoonosis Science Center, Uppsala University, Uppsala, Sweden; c St. Giles Laboratory of Human Genetics of Infectious Diseases, The Rockefeller University, New York, New York, USA; d Department of Microbiology and Immunology, New York Medical College, Valhalla, New York, USA; e Departmento de Medicina, Universidad del Norte, Barranquilla, Colombia; f London School of Hygiene and Tropical Medicine, London, United Kingdom; g Pôle de Recherche et de Veille sur les Maladies Infectieuses Émergentes, Institut Louis Malardé, Tahiti, French Polynesia; h UMR Emergence des Pathologies Virales (EVP), Aix-Marseille Université, IRD 190, INSERM 1207, EHESP, Marseille, France; i Institut Hospitalo-Universitaire Méditerranée Infection, APHM Public Hospitals of Marseille, Marseille, France

## ERRATUM

Volume 7, issue 5, e01239-16, 2016, https://doi:10.1128/mBio.01239-16. The following information should have been included in Acknowledgments.

The funders had no role in study design, data collection and interpretation, or the decision to submit the work for publication.

J.H.-O.P., V.E., D.M., A.N., R.C., E.A.G., and X.D.L. are members of the European Union’s Horizon 2020 research and innovation program ZIKAlliance under grant agreement 734548, and A.K.F. and M.W.G. are members of ZikaPLAN under grant agreement 734584.

