# Supplementary figures and images for: How Did Zika Virus Emerge in the Pacific Islands and Latin America?
Source: mBio. 2016 Oct 11;7(5):e01239-16. doi: 10.1128/mBio.01239-16 (PMC5061869; doi:10.1128/mBio.01239-16)

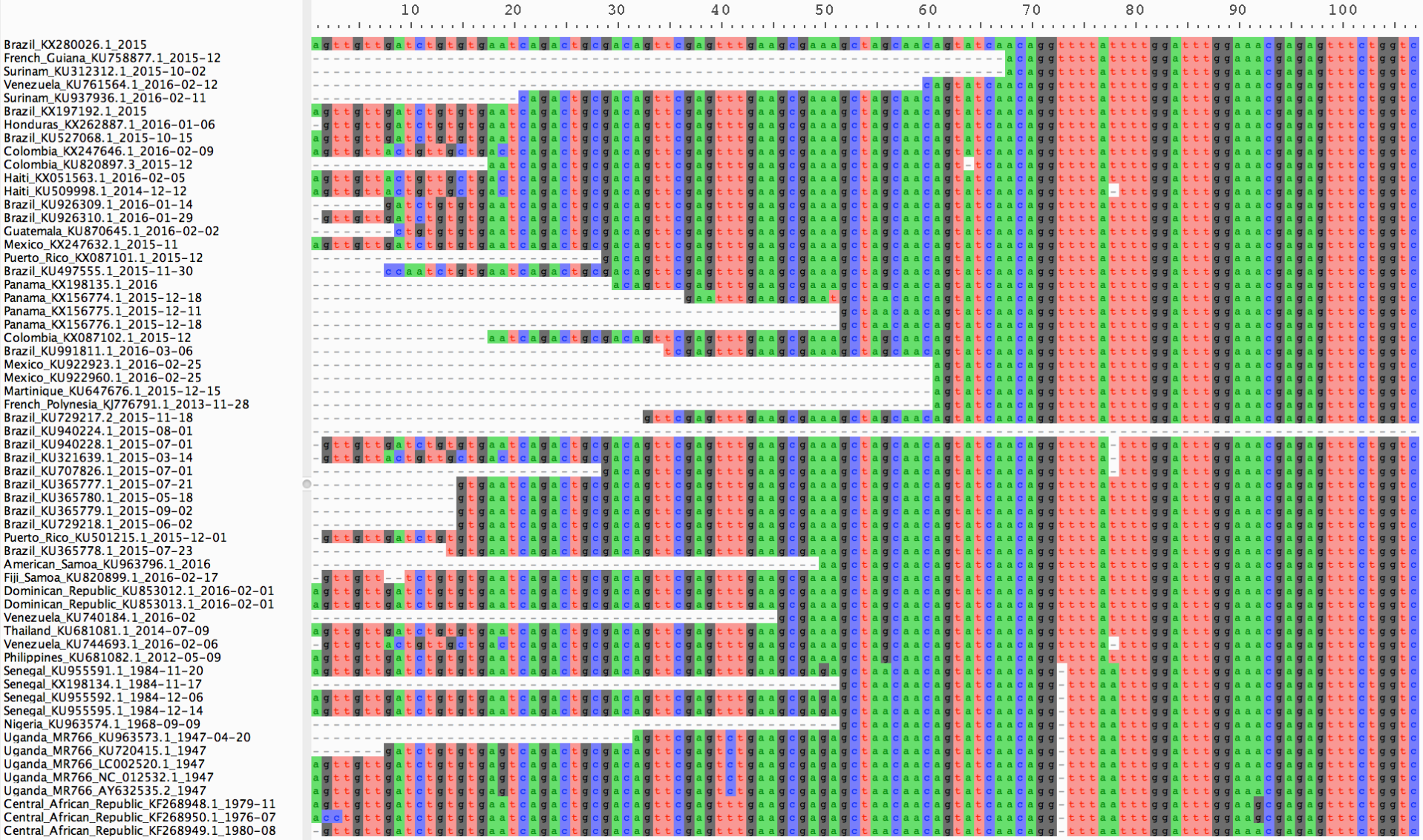

Supplement: Figure S1 — Alignment of 5′ UTRs of Zika virus isolates in PDF format. Download [file mbo005163018sf1.pdf]

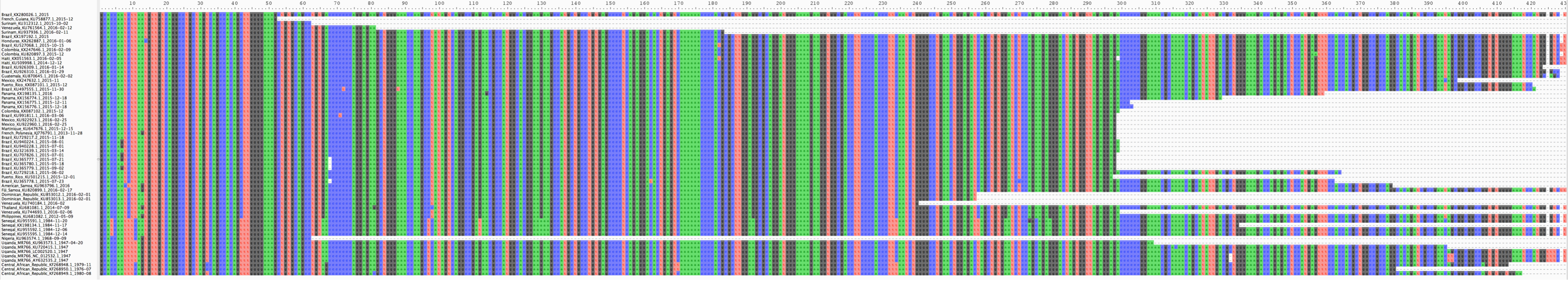

Supplement: Figure S2 — Alignment of 3′ UTRs of Zika virus isolates in PDF format. Download [file mbo005163018sf2.pdf]
